# What is palliative care? Perceptions of healthcare professionals

**DOI:** 10.1111/scs.12603

**Published:** 2018-08-13

**Authors:** Birgitta Wallerstedt, Eva Benzein, Kristina Schildmeijer, Anna Sandgren

**Affiliations:** ^1^ Department of Health and Caring Sciences Faculty of Health and Life Sciences Linnaeus University Sweden; ^2^ Center for Collaborative Palliative Care Linnaeus University Sweden

**Keywords:** palliative care, perceptions, healthcare professionals, focus group interviews, conceptual definition, communication, collaboration

## Abstract

**Background:**

Despite increased attention and knowledge in palliative care, there is still confusion concerning how to interpret the concept of palliative care and implement it in practice. This can result in difficulties for healthcare professionals in identifying patients whom would benefit from palliative care, which, in turn, could lead to a delay in meeting patients’ needs.

**Aim:**

To explore healthcare professionals’ perceptions of palliative care.

**Method:**

Data were collected through twelve interprofessional focus group interviews in community care and hospital wards in south Sweden (n = 74). All interviews were analysed with latent content analysis.

**Results:**

Three domains were revealed: first, *a blurred conceptual understanding* as participants described palliative care using synonyms, diagnoses, phases, natural care and holism; second, *a challenge to communicate transitions* concerned the importance of how and when the transition to palliative care was communicated and documented; finally, *a need for interprofessional collaboration* was described as well as the consequences for severely ill persons, relatives and healthcare professionals when it was not established.

**Conclusion:**

The perceptions about how to interpret palliative care differed as well as when palliative care should be offered and decided, which might have practical consequences. How long a person has left to live is of great significance for decision‐making, caregiving and preparation in palliative care. The challenge is to use interprofessional communication to promote understanding and collaborate across varied care levels. Integrating palliative care across diverse care levels could be one way to reduce the ambiguity of palliative care.

## Introduction

The World Health Organization (WHO) defines palliative care as an approach that focuses on improving the quality of care of seriously ill persons and their families 1. Although attention and knowledge about palliative care during the last decade have increased among healthcare professionals, studies show there is still confusion concerning how to interpret the concept of palliative care and implement it in practice 2, 3. For example, healthcare professionals may find it difficult to identify patients whom would benefit from palliative care 4, 5, and this difficulty may lead to delays in meeting patients’ needs 6. Earlier studies have also noted challenges for healthcare professionals when providing palliative care including dealing with moral conflicts between what they can deliver versus what they would like to provide 7, 8, the impact of physicians’ attitudes on the decided treatments 8, 9, communication difficulties among healthcare professionals 9, 10 and poor cooperation between healthcare professionals 11. To provide high‐quality care based on patients’ and relatives’ needs, interprofessional collaboration based on a shared view of the essence of palliative care is a prerequisite 12, 13. Although some studies have addressed healthcare professionals’ experiences working in palliative care, studies on their own perceptions of the practice of palliative care have rarely been conducted.

### Aim

The aim was to explore healthcare professionals’ perceptions of palliative care.

## Methods

### Design

The study employed a qualitative design using focus group interviews 14.

### Setting and sample

The study was conducted in several municipalities from the same county in south Sweden. Locations comprised wards (cardiological, gynaecological and oncological care) at a middle‐sized hospital, nursing homes (including short‐term care) and home healthcare settings. All locations had daytime‐limited access to a palliative advisory team.

The recruitment of the professionals was made by each manager, who purposefully gave oral and written information about the study to healthcare professionals and invited them to participate. Our intention was to obtain a sample with a variety of age, sex, profession and length of working in palliative care. Those who were interested to participate informed the manager who sent the information further to the researchers. All professionals who were asked agreed to participate. At the beginning of each interview session, the participants were invited to ask questions whether anything was unclear about their participation. When no questions were left, they filled in the written informed consent sheet. Twelve interprofessional focus group interviews were performed between 2014 and 2016; seven in community care wards and five in hospital wards. All participants simultaneously cared for patients with palliative care needs and patients with curable diseases at their workplace, except for two, who worked in a palliative advisory team at the hospital. The composition of the groups is presented in Table 1.

**Table 1:** Focus groups composition

Setting	Total (men/women) n = 74 (5/65)	Assistant nurses n = 32	Managers n = 7	Nurses n = 26	Paramedics n = 4	Physicians n = 2
1 Community care	7 (1/6)	2	3	1	1	0
2 Community care	7 (1/6)	7	0	0	0	0
3 Community care	8 (1/7)	7	0	1	0	0
4 Community care	6 (0/6)	3	1	2	0	0
5 Community care	5 (0/5)	1	2	1	1	0
6 Community care	5 (0/5)	2	1	2	0	0
7 Community care	6 (0/6)	2	0	2	2	0
8 Cardiology ward	5 (1/4)	0	1	3	0	1
9 Gynaecologic ward	6 (0/6)	2	0	4	0	0
10 Gynaecologic ward	7 (0/7)	3	0	4	0	0
11 Oncology ward	6 (1/5)	0	0	5	0	1
12 Oncology ward	6 (0/6)	3	0	3	0	0

### Data collection: focus groups

The intent of the focus groups was to capture participants’ perceptions, feelings and thoughts about a specific topic, by promoting self‐disclosure among participants. The group interactions may facilitate participants to explore and clarify their attitudes and values 15. The unique possibility is the capacity ‘to become more than the sum of its parts’ (14, p. 19). In other words, participants co‐create the data in a synergetic way.

One moderator and one assistant moderator performed the interviews: the moderator having the main responsibility for the content, to encourage comments and to facilitate interaction between the participants while the assistant moderator handled the practical issues, such as time constraints 14. The constellation of moderators varied, and except for the first, second and last author, two further researchers acted as moderators. The tape‐recorded interviews lasted between 60 and 90 minutes and were conducted using an interview guide with open‐ended questions. To encourage participants to reflect on the topic, the interviews began with a general question, ‘*When you hear “palliative care,” what do you think of*?’ As the interview proceeded, the questions became increasingly focused (e.g. ‘*How do you want to describe the palliative care approach?*’). The moderator also asked questions to facilitate interaction between participants (e.g. ‘*When you hear your colleague express his/her thoughts like that, what do you think then*?’ or ‘*What are your reflections of what you just heard*?’). These kinds of questions promoted conversation. In addition, questions to clarify or deepen the answers were posed (e.g. ‘*Can you tell us more about that?*’). The interaction between the moderators and the participants made the conversational climate comfortable.

### Analyses

All interviews were considered as one data set and were analysed with latent content analysis as discussed by Graneheim and Lundman 16. In the first step, three of the authors (BW, AS and EB) read the whole text to get a sense of the whole and to identify common issues across the interviews. Second, the first author performed the coding (i.e. placing similar labels on parts of the text containing similar content areas). Third, content areas were categorised and findings were discussed and refined after a peer‐reviewed seminar. Finally, the categories were reflected on and interpreted into domains. The results were then discussed for credibility, and consensus was reached by all authors.

## Results

The results revealed three domains: *a blurred conceptual understanding, a challenge communicating transitions* and *a need for interprofessional collaboration*.

### A blurred conceptual understanding

The results revealed various perceptions about palliative care; some were shared and some differed within the groups and between healthcare professionals. Overall, palliative care was expressed as a blurred and confusing concept. When talking about palliative care, the participants used various synonyms. The immediate understanding was that palliative care was equivalent with end‐of‐life care; however, delving further into the conversations revealed that this perception was altered in a more nuanced way, including thoughts that palliative care did not only concern the final phase of life. However, simultaneously, participants also used terms such as ‘terminal care’ and ‘last time in life’, making the concept even further confusing. In addition, some professionals from community care claimed that the term ‘palliative care’ was not often used daily in their care context. Instead, ‘caregiving close to death’ was referred to as a natural part of life, when the ill person gradually deteriorated and finally died. In that phase of life, doing ‘the little extra’ was important in caregiving. Others described palliative care as a downward process for days, months or years. This quote exemplifies participants’ thoughts about palliative care:

Respondent: Yes, maybe the last weeks.

Respondent: The last weeks.

Interviewer: Do all of you think so?

Respondent: I think a little longer, palliative care …

Respondent: Me too.

Respondent: …That it's for very sick people but it can also last for many months or …

Respondent: At least one year, maybe, a bit different.

Respondent: And I say the same, chronically ill people with diseases that they may live with for many years.

(Focus group interview 5)

A common and shared perception was that palliative care covered a holistic view of the care, including physical, psychological, social and existential aspects. This view also included relatives as an obvious and natural part in palliative care. The participants acknowledged they played a key role; however, their participation varied from constantly being with the dying person to making a few visits at critical times.

Another part of participants’ understanding of palliative care was the link with special diagnoses. Although cancer is commonly linked to palliative care, other diseases such as chronic obstructive lung diseases, dementia and heart failure are increasingly common. Palliative care was also understood using palliative phases; however, understanding what phase one is in varied in clarity, related to when palliative care should be used in practice. In early phases, participants perceived that the ill person often seemed to feel well and may be able to stay at home. However, later phases were interpreted as a time of deterioration, when symptoms and fatigue increased in ill persons.

### A challenge to communicate transitions

Participants’ perceptions of palliative care were also connected to how and when the transition to palliative care was communicated. For example, as the meaning of palliative care had changed over time, it was not easy for the participants to determine when the transition from curable treatment to palliative care was occurring. Palliative care also includes an increasing number of treatment lines, making the transition points even more unclear. These treatments could be offered for a long time, and persons with advanced diseases can live many years. The participants also argued that there was a risk of ill persons to be ‘treated to death’, thereby losing the dignity.

It was discussed that having these kinds of seriously illness conversations with patients and their families acted a barrier for many physicians. Some did not hesitate at all; they handled this task in a professional way, especially physicians from the palliative advisory team. Others seemed to have great difficulties and postponed or even handed over the task to the nurses. Sometimes these conversations were not held at all, often when a hired doctor was involved.

Respondent: Now, sometimes they have a conversation about serious illness even with family members … that now we cannot cure you.

Respondent: No.

Respondent: But I wish it was clearer sometimes.

Respondent: Many doctors do not want to conduct them, only some do.

Respondent: Yes, sometimes you feel that it's also hard for a doctor to engage in those conversations. We had some confusion with relatives who did not really think it was as serious as it was. It is actually a patient we now have in a palliative phase… where the relative did not realize that…

Respondent: No.

Respondent: … despite repeated conversations. Then one can question … the clarity.

(Focus group interview 9).

The time when serious illness conversations were performed was also discussed (i.e. if it was done early, right on time or too late). The participants argued that the timing of the conversations heavily impacted the caring situation. A common perception was that these conversations should be performed early in the illness trajectory and repeated alongside with ongoing treatment lines. This opinion was especially emphasised by the participating physicians. A fluctuating disease course made it harder to make these decisions, compared to a more predictable prognosis. The main reason to have these conversations in a timely fashion was to promote quality of life in the ill person before they died. However, there were many discussions about difficulties in knowing when it was the right time to initiate these conversations. It should, according to the participants, be related to the readiness of the ill person and his/her family members. When the ill person and the physician knew each other well, it was much easier to have these conversations and make necessary decisions. When physicians were afraid or hesitated to initiate these conversations, it was often too late. This was emotional for many participants, and they clearly expressed their frustration about the consequences for the ill persons, the ill persons’ relatives and for themselves.

Further, these conversations were often poorly documented, and healthcare providers had to ‘read between the lines’ to know what had been said. The outcome of the conversations was also poorly communicated to involved parties. Assistant nurses, the staff group who often are closest to the ill person, expressed that they had never participated in these conversations. Often, they did not know whether these conversations had been conducted nor what was said. The participating physicians, on the other hand, indicated that these conversations were accurately documented in their workplace and that they sometimes communicated with other professionals involved in the care.

### A need for interprofessional collaboration

Interprofessional collaboration was described as important in palliative care to increase the ill person's well‐being and to make the death as easy as possible. The participants noted that the care delivered to the ill person should be characterised by preserving dignity, which is facilitated if everybody is working in the same direction. However, it was said that this collaboration in palliative care could be improved using proactive, diverse professional knowledge earlier in the process.

In some care contexts, diverse team meetings were performed regularly concerning how to manage the care of severely ill persons. It was argued that these meetings needed to be performed more frequently and be related to fast changes and progresses in the illness trajectory. If there was a long time between these meetings, there was a risk of a noncollaborative care culture to appear, where each professional creates his/her own approach.

The palliative advisory teams were experienced as a very important resource in performing palliative care. They supported transition communication, treatment and care between involved parties and performed serious illness conversations with patients and their families. Their involvement with information, knowledge and continuity created security in palliative care. On some occasions, when care delivery was especially strenuous, supportive and supervising collaboration with the church or a counsellor was used.

Sometimes the transition to palliative care was a joint decision between several physicians, especially if patients’ condition fluctuated over time. Other forms of collaboration included when nurses initiated discussions with physicians about a deteriorated patient's need for palliative care, often based on the observations of assistant nurses. It was obvious that the team constellation typically consisted of either nurses and a physician or a nurse and assistant nurses. The team was only completed with other professionals occasionally when a care problem needed to be solved.

Many participants highlighted the need for increased collaboration and communication between the nurse and nurse assistants. There were also opinions that the collaboration between home health care and hospital wards left much to be desired. Nurses’ need for hunting knowledge, about decisions made concerning patients, was described as very unsatisfactory. Knowing that the focus was palliative care was important because they knew what to expect and could prepare how to discuss key issues with patients and their families. Not knowing the focus, due to a lack of or poorly performed conversation documentation, affected their security in professional practice. When deterioration occurs, the participants argued for the need to know what to do, which was linked to decisions and communication. In these situations, it was described that the patient and their relatives clearly noticed how healthcare professionals behaved and expressed themselves. When timing is an issue, it is especially crucial for healthcare providers to know the care focus to avoid ambiguity and create an individualised and safe care relationship with dying persons.

Respondent: No, I'm just thinking about this … that you can generally be better in communicating and sharing experiences, for everyone to join the same track, and yes, to have a goal that you work towards…

Respondent: Additionally, you may have a meeting…afterwards and talk about if the care process was good or not. When the ill person has passed away, we move on, and then there will be new ones. It's rarely…

Interviewer: …Opportunity to reflect?

Respondent: Yes, afterwards.

(Focus group interview 7).

## Discussion

The conceptual understanding of palliative care was perceived by the participants in an ambiguous and blurred way, reflecting the complexity of the concept. This was highlighted as a problem fifteen years ago 17 and confirmed in subsequent research 2, 3. In addition, similar concepts require clarification (e.g. ‘a palliative approach’ 18. Typically, palliative care was identified as ‘end‐of‐life care’, but also as ‘terminal care’, ‘last time in life’, ‘natural care’ and so on. Previous research confirms that palliative care is often interpreted as end‐of‐life care 19. This understanding of palliative care also has an impact on the care provided, as previous studies have found that a blurred understanding of palliative care contributes to inadequate 20 and underutilised care of people with palliative care needs 19.

Our results also stress that education level might influence the understanding of how to interpret palliative care. Indeed, most participants had no special education or training within palliative care, which seems to be a concern among healthcare professionals in Sweden 21, 22, except for those working in specialised palliative care units. This situation has been highlighted as a prioritised area both in Swedish 23 and European policy documents 24.

The results also show ambiguity about when the transition from cure to palliative care occurs and when it should be communicated. This ambiguity was related to the blurred boundaries between curative and palliative care. Several factors may influence when curative care is abandoned in favour of palliative care such as agreement, timing and decision‐making 25. In a survey of over 800 physicians and nurses, respondents argued that decisions to start palliative care were made too late. This was also emphasised in our study. Obviously, a too late transition to palliative care is not beneficial for patients, their family members or staff. This renders the question, is quality of care jeopardised due to a late or a too late transition to palliative care? In a retrospective cohort study 26, it was addressed how the timing and setting of palliative care referral were associated with the quality of end‐of‐life care. The results showed that earlier palliative care referral was associated with fewer emergency room visits, hospitalisations and hospital deaths for outpatients in the last month of life. Hui and colleagues concluded the need for early integration of palliative care 26, as did Scibetta and colleagues 27, who compared healthcare utilisation and care quality for deceased cancer patients who received early versus late palliative care. In addition, early palliative care is associated with less intensive medical care, improved quality outcomes and cost savings. However, despite recommendations about early palliative care, these services remain underutilised. We concur with O'Shea 28 that there is a need to shift to more integrated palliative care, such as the suggestion made from the WHO 29 (e.g. to improve care quality by integrating palliative care in health system policies at all levels of care).

The timing of conversations about serious illnesses and how to perform them were highlighted as important in our study. Although it was argued that some physicians performed the conversations without hesitation, the perception was that many physicians did postpone or avoided these conversations. Ho and colleagues 30 found that physicians felt uncomfortable with death and dying and perceived that other professionals were better trained to conduct these conversations. This is in line with a recent study involving dialysis care physicians 31. Reasons for hesitation were that they felt uncertain about their skills to handle the conversations. These studies showed that physicians would benefit from training and coaching in how to perform difficult conversations. There are, however, diverse ways to perform these conversations; for example, Bernacki and colleagues 32 described a model for training conversations between the seriously ill person and physicians aimed to be performed more often, earlier and more effectively.

Interprofessional collaboration was seen as important for delivering quality palliative care. Lloyd and colleagues 13 argued that multidisciplinary team collaboration is a prerequisite and that multidimensional skills in the team are resources that can help identify for persons in need of palliative care. Further, Siouta and colleagues 33 identified models of palliative care in cancer and chronic disease aiming for integrating palliative care in Europe. Involvement of a palliative care multidisciplinary team resulted in better symptom control, less caregiver burden, improved continuity, improved coordination of care, fewer admissions, cost‐effectiveness and ensuring that patients died in their preferred place.

Approaching palliative care as an interprofessional task to meet care needs in a holistic way is also supported by the WHO 1. Our results showed, however, that healthcare teams consisted mostly of two professionals working together with the occasional assistance of other professionals. This mirrors a more traditional care culture, where each professional act on their own without having a dialogue with others. However, the importance of communication between involved parties was highlighted many times in our study, mostly in relation to forming a consensus about how to understand the meaning of palliative care. According to Klarare and colleagues 34, communication is key for team effectiveness, resolving conflict and executing palliative care. This is supported by Pype and colleagues 35, who also highlighted the importance of competence among team members, team arrangements and task descriptions. However, many participants in our study neither worked nor communicated as an interprofessional team. Instead, nurses acted as communicators between diverse professionals and care levels, and nurse assistants were sometimes at risk of losing vital information. Liaschenko and colleagues 36 confirm nurses’ role as mediating palliative care to other disciplines, obtaining information from various sources and synthesising and using the information to develop a holistic assessment. Moreover, Bainbridge and colleagues 37 argued that palliative networking can facilitate interprofessional communication. We agree with Lloyd and colleagues 13 that interprofessional collaboration is a prerequisite for high‐quality palliative care where palliative knowledge and experiences are transferred across disciplines.

### Study strengths and limitations

There are some strengths and limitations to be considered when using focus groups 14, 38 and examining interprofessional perceptions when caring for terminally ill patients 15, 39. We argue that our data collection procedure provided rich data in a rather limited amount of time. Participants had the possibility to interact directly with other participants both verbally and nonverbally, and data were revealed in a synergistic way as participants could build on the responses from each other. We tried to facilitate a welcoming atmosphere, as proposed by Stewart & Shamdasani 38, where participants felt comfortable, respected and nonjudged. This scenario is highly related to the skills of the moderators, who all had vast experience in conducting group interviews, had interpersonal skills and a contextual understanding of palliative care 39. However, the use of multiple moderators in our study may have jeopardised the reliability of the results 40; nonetheless, we argue the interviews were performed in a comparable way using the interview guide. All moderators adapted a directive interview style, being most active at the beginning of the interview and later focusing on keeping the discussion on track.

The groups included six to eight participants each. There is no consensus about maximum and minimum numbers of participants in groups; however, 5–10 14 or 8–12 people 38 are often recommended. In a palliative care context, 7–10 people have been proposed 39. Most importantly, the group must be small enough to ensure everyone can share their insights, but large enough to allow for diverse perceptions 14. We argue that we fulfilled these requirements. Further, the multiprofessional composition of the groups includes diverse ages and both sexes, which promoted multiple perspectives in the dialogues. However, most of the participants were women, who mirror the sex distribution of Swedish healthcare staff. A potential limitation is the few numbers of participating physicians, as it would have been preferable to have one in each group.

## Conclusion

It is obvious that the current ambiguous understanding of palliative care has a negative impact on the care that is provided to patients and the emotions of family members and healthcare professionals. We strongly argue that a common conceptualisation would enhance care, interprofessional communication and teamwork. However, this is not sufficient to meet the needs of people in need of palliative care. There is also an urgent need for education and training in palliative care for healthcare professionals. Further research is needed regarding whether a more integrated understanding of the concept palliative care may improve the quality of palliative care.

## Clinical implications

- Start a dialogue in interprofessional groups about how to interpret, understand and communicate the meaning of palliative care that is common across units and care levels.
- At a leadership level, facilitate palliative care education and training for all healthcare staff.
- Provide tailored education for physicians and other professionals who must initiate and perform difficult conversations with severely ill individuals and their families.

## Author contributions

The study design was implemented by EB, AS and BW. All authors conducted interviews; BW initially analysed the data, which then were discussed several times by all authors to reach consensus. AS and KS wrote the introduction, EB was responsible for method and method discussion sections, BW wrote the results and all authors collaborated in the discussion. BW drafted the manuscript; however, all authors took responsibility for revising the final manuscript.

## Ethical approval

As no ethical approval is required in Sweden for research involving healthcare professionals, the whole research process was guided by the ethical principles for medical research (the Declaration of Helsinki). The COREQ guidelines 41 were used for reporting our research.

## Conflict of interest

All authors declare no conflict of interests in connection with this article.

## Funding

This research was supported by the Kamprad Family Foundation for Entrepreneurship, Research & Charity.
